# Targeting STAT6 in PMBL

**DOI:** 10.18632/oncotarget.2447

**Published:** 2014-09-05

**Authors:** Karen Leroy, Anais Pujals, Laura Pelletier

**Affiliations:** INSERM U955, Université Paris Est Creteil, Département de Pathologie, Hôpital Henri Mondor, Créteil, France

Primary Mediastinal B-cell Lymphoma (PMBL) is a specific subtype of B-cell lymphoma which shares some phenotypic and genotypic features with diffuse large B-cell lymphoma (DLBCL) and with classical Hodgkin's lymphoma (HL). The genomic landscape of PMBL has not been fully elucidated, but has revealed the recurrent alteration of genes involved in JAK-STAT signaling and in immune surveillance [1]. The activation of JAK-STAT signaling pathway has been linked to recurrent mutations of *SOCS1* or *PTPN1* [2] and to *JAK2* amplifications [3]. Mutations of the *STAT6* gene in the exons encoding the DNA binding domain are detected in about 35% of the cases and in MedB-1 PMBL derived cell line, but their functional consequences are so far unknown [4]. Curiously, genetic alterations of *SOCS1, PTPN1, JAK2* and *STAT6* overlap in some tumors, suggesting that their effects may not be strictly redundant.

The optimal treatment strategy of PMBL remains to be precisely defined. The results of a recent trial indicate that dose intensive chemotherapy (dose adjusted EPOCH) combined with rituximab may be the best option nowadays, limiting the need of consolidation radiotherapy and the risk of associated sequelae [5]. An alternative option could be to associate standard chemotherapy with targeted therapy, as suggested by the results reported by O Ritz and colleagues [6]. In this study, the authors showed that transient STAT6 knockdown with a siRNA, which induces no cell death on its own, strongly sensitizes Karpas1106, a PMBL derived cell line, to doxorubicin, vincristin or rituximab. STAT6 knockdown induced some cell death in MedB-1 cells, but did not affect the response of the cells to these compounds. A third PMBL cell line, U2940, showed an intermediate phenotype: a decreased viability upon STAT6 knockdown at the basal level and upon doxorubicin treatment. The authors also used a siRNA targeting BCL6 oncogene, which is expressed in PMBL cell lines with a mutually exclusive pattern with phospho-STAT6. Karpas1106 and U2940 cell lines proved sensitive to BCL6 downregulation, but this sensitivity did not notably impact the response to the drugs. Overall, these results suggest that some PMBL cells could benefit from treatments associating a targeted therapy against JAK-STAT signaling and standard immuno-chemotherapy. Interestingly, O Ritz and colleagues had previously shown that PMBL cell lines are sensitive to JAK2 inhibition [7]. Moreover, clinical grade specific inhibitors of JAK2, such as fedratinib, were recently shown to decrease HL and PMBL cell growth *in vitro* and *in vivo*, and to induce a broad inhibition of STAT proteins phosphorylation [8]. Hence, upstream inhibition of the pathway may prove quite efficient to block STAT activity and increase sensitivity to chemotherapy in these cells.

The response of cell lines cultured *in vitro* to specific inhibitors is a strong indicator that the targeted pathway is biologically relevant. Nevertheless, a lot of questions still remain: which target will be the most efficient to kill tumor cells without major side effects: JAK2 or STAT6? Why does STAT6 knock-down affect drug sensitivity in some cell lines but not in others: is it related to the presence of *STAT6* or any other gene mutations? Should these targeted therapies be given alone, or in association with immuno-chemotherapy? And will these strategies prove more efficient than dose adjusted EPOCH-rituximab? Last but not least, as mentioned previously, PMBL are also characterized by the alteration of the immune surveillance system. It would be worth evaluating the relationship between JAK-STAT activation and the immune privilege of these tumors, because it could impact patients' outcome upon targeted therapy.
